# Serine phosphorylation of the cotton cytosolic pyruvate kinase GhPK6 decreases its stability and activity

**DOI:** 10.1002/2211-5463.12179

**Published:** 2017-01-25

**Authors:** Bing Zhang, Jin‐Yuan Liu

**Affiliations:** ^1^ Laboratory of Plant Molecular BiologyCenter for Plant BiologySchool of Life SciencesTsinghua UniversityBeijingChina; ^2^ Tsinghua‐Peking Center for Life ScienceTsinghua UniversityBeijingChina

**Keywords:** cotton fiber, fast cell growth, phosphorylation, pyruvate kinase, ubiquitination

## Abstract

Pyruvate kinase (PK, EC 2.7.1.40) is an important glycolytic enzyme involved in multiple physiological and developmental processes. In this study, we demonstrated that cotton cytosolic pyruvate kinase 6 (GhPK6) was phosphorylated at serines 215 and 402. Phosphorylation of GhPK6 at serine 215 inhibited its enzyme activity, whereas phosphorylation at both serine sites could promote its degradation. The phosphorylation‐mediated ubiquitination of GhPK6 was gradually attenuated during the cotton fiber elongation process, which sufficiently explained the increase in the protein/mRNA ratios. These results collectively provided experimental evidence that cotton fiber elongation might be regulated at the post‐translational level.

**Abbreviations**
EMM: Edinburgh minimal medium
MALDI‐TOF: matrix‐assisted laser desorption/ionization‐time of flight
PEPC: phosphoenolpyruvate carboxylase
PEP: phosphoenolpyruvate
PK: pyruvate kinase
ROS: reactive oxygen species

Cotton is one of the most important economical crops cultivated globally. After undergoing a period of fast elongation (~ 20 days) and a secondary wall deposition (~ 30 days), trichomes initiated from cotton ovule epidermal cells develop into spinnable fibers, which is a great textile material with many applications 1. The cotton fiber cell is one of the longest and fastest elongating cells in the plant kingdom, making it a suitable model system for studying the mechanism of fast cell expansion 2. In recent years, the key roles of carbohydrate metabolism in cotton fiber development have gradually been recognized. Specifically, enzymes involved in carbohydrate metabolism including sucrose synthase, invertase, and phosphoenolpyruvate carboxylase were all found to play important roles in the development of cotton fibers 3, 4, 5. However, the detailed molecular mechanisms regarding how carbohydrate metabolism in cotton fiber cells is regulated, especially at the post‐translational level, are still poorly understood.

As an important glycolytic enzyme, pyruvate kinase (PK) catalyzes the transfer of a high‐energy phosphate group from phosphoenolpyruvate to ADP to produce ATP and pyruvate. Pyruvate then enters the mitochondria to generate large amounts of ATP through the tricarboxylic acid (TCA) cycle with oxidative phosphorylation. Because glycolysis and the TCA cycle are at the center of metabolism, regulating PK activity is important for controlled growth and development of both animals and plants 6. In mammals, PK activity is regulated at multiple levels. Different PK isoforms are produced through alternative splicing in different tissues 7, whereas post‐translational modifications including phosphorylation, acetylation, and oxidation are applied to different PK isoforms to further alter their abundance and enzymatic activities 8, 9, 10.

In plants, PKs exist as plastid and cytosolic isozymes. Plastid PK (PKp) isozymes are mainly involved in seed oil biosynthesis 11, 12, whereas cytosolic PK (PKc) isozymes participate in the growth and developmental regulation at both the organic and cellular levels 13, 14, 15. Similar to their mammalian orthologs, plant PKc isozymes are also regulated by post‐translational modifications. For example, the 55‐kDa soybean PKc isozyme is phosphorylated at serines 220 and 407 16. C‐terminal proteolytic processing of the 55 kDa PKc isozyme could further generate a truncated 51 kDa protein 16. The influence of these two post‐translational modifications on soybean PKc protein abundance was successfully determined; however, the physiological function of these two modifications are still unclear.

In our previous study, we have revealed that cotton cytosolic PK GhPK6 is differentially expressed in elongating cotton fibers and participates in fast fiber elongation regulation in a reactive oxygen species (ROS)‐mediated manner 15. In this study, we further elucidated that GhPK6 was phosphorylated at serine residues 215 and 402, similar to the soybean PKc. Phosphorylation of GhPK6 at the two conserved serine sites significantly promoted its ubiquitin‐dependent degradation, which explained the observed protein/mRNA ratio change of GhPK6 in elongating cotton fibers 15. The results from this study greatly improve our understanding of metabolic regulation during the cotton fiber elongation process.

## Materials and methods

### Plant materials

Upland cotton (*Gossypium hirsutum* L.) cultivar CRI 35 was grown in a normal agronomic field at Tsinghua yuan, Beijing. Cotton flowers on the day of anthesis (0 dpa) were tagged, and the developing cotton bolls were harvested during various stages. The fibers were carefully dissected from each boll, immediately frozen in liquid nitrogen, and stored at −80 °C until further use for protein extraction.

### Amino sequence alignment and structure prediction

The amino acid sequences of PK proteins from different organisms were aligned using clustalx (version 2.1) (http://www.ch.embnet.org/software/ClustalW.html). The 3D protein structure of GhPK6 was modeled using the automated mode of the SWISS‐MODEL server (http://swissmodel.expasy.org/). The structure was displayed using viewerlite (version 5.0) (http://viewerlite.software.informer.com/).

### Site‐specific mutation, protein expression, and purification

Site‐specific mutations of the GhPK6 ORF were generated using the overlap PCR method 17. The full‐length ORF of wild‐type (wt) and mutated GhPK6 were amplified by PCR, digested with *Bam*HI and *Sac*I, and cloned into the prokaryotic expression vector pET‐28a (Novagen, Madison, WI, USA). The primers are listed in Table S1. The recombinant His‐tagged proteins were expressed in the *Escherichia coli* strain BL21 (DE3) and purified using a nickel sulfate resin following the manufacturer's instructions (Qiagen, Hilden, Germany).

### Enzyme activity assay

The enzymatic activity of the recombinant His‐tagged proteins was determined as previously described 18. Briefly, 1 μg of purified recombinant protein was added into 500 μL of assay solution containing 50 mm HEPES‐KOH (pH 6.4), 25 mm KCl, 12 mm MgCl_2_, 2 mm phosphoenolpyruvate, 1 mm ADP, 1 mm DTT, 5% (w/v) PEG8000, 0.15 mm NADH, and 2 units·mL^−1^ lactate dehydrogenase (Sigma, Shanghai, China). The reaction was coupled with the lactate dehydrogenase reaction and assayed at 25 °C by monitoring the oxidation of NADH at 340 nm using an Ultrospec 3300 Pro spectrophotometer (Amersham Biosciences, Uppsala, Sweden) with a continuous recording function. The *V*_max_ values were automatically calculated by the spectrophotometer.

### Fission yeast expression

The ORF of wt and mutated GhPK6 were amplified by PCR, digested with *Bam*HI and *Sac*I, and cloned into the pESP2M vector. The vector was then transformed into *Schizosaccharomyces pombe* SP‐Q01 cells by the LiCl‐PEG method 19. The transformants were selected on plates containing minimal medium (EMM) that was supplemented with 75 mg·L^−1^ adenine and uracil and grown at 32 °C. Thiamine was added to the medium (final concentration of 2 μm) to repress the NMT1 promoter 20. To induce protein expression, the PCR‐confirmed positive colonies were first grown in liquid EMM with thiamine supplementation until the midexponential phase and then washed three times using EMM without thiamine to release the promoter repression.

### Antibody preparation and western blotting

Specific antibodies were prepared by immunizing rabbits with synthesized protein‐specific and BSA‐coated phosphorylation site‐specific peptides mixed with Freund adjuvant by Beijing Protein Innovation (Beijing, China) and the phosphoserine/threonine/tyrosine antibody was purchased from Abcam (ab15556, Hong Kong, China) (Table S2). Two‐step antigen affinity purifications were further performed to enrich for phosphorylation site‐specific antibodies to ensure the titers against BSA‐coated synthesized phosphorylation site‐specific peptides were larger than 1 : 12 800 by ELISA (Fig. S1). Goat anti‐rabbit and goat anti‐mouse antibodies that were conjugated to horseradish peroxidase were used as secondary antibodies (Abcam, ab97051 and ab97023, respectively). For western blotting, 20 μg of protein for each sample was denatured in 6 × SDS/PAGE sampling buffer by boiling in a water bath for 5 min, separated by 12% SDS/PAGE and transferred to a PVDF membrane. The signals were developed with a Lumi‐Light western blotting substrate (Roche, Mannheim, Germany).

### Immunoprecipitation

For the immunoprecipitation, total protein was first extracted as previously described 21. Approximately 1 g of frozen cotton fibers was ground in a chilled mortar and pestle with 0.1 g of quartz sand, 0.1 g of PVPP, and 1.5 mL of IP buffer [50 mm Tris‐HCl pH 7.5, 150 mm NaCl, 10 mm MgCl_2_, 1 mm PMSF, 1% (v/w) NP‐40, 100 × Protease Inhibitor Cocktail (Sigma, P2714), and 100 μm MG132 (Sigma, C2211)]. The sample was spun at 16 000 ***g*** for 10 min at 4 °C to remove the cellular debris. After reserving 40 μL of the supernatant as input, the remaining supernatant was transferred to a tube containing 10 μL of anti‐GhPK6 antibody and 50 μL of Protein‐A beads (Santa Cruz, Texas, USA) and incubated for 4 h at 4 °C with gentle rotation. After incubation, the beads were spun at 1500 ***g*** for 30 s at 4 °C. The beads were washed thoroughly three times with 1 mL of IP buffer before adding 40 μL of 6 × SDS/PAGE sampling buffer. Then, the samples were boiled, separated by 12% SDS/PAGE and examined by western blotting using an anti‐ubiquitin antibody (ab7780; Abcam).

### *In vitro* kinase assay

The *in vitro* kinase assay was performed according to the method as previously described 22. Briefly, 5 μg of the purified wt and mutant GhPK6 proteins were incubated in 50 μL of phosphorylation buffer [50 mm Tris‐HCl (pH 8.0), 100 mm NaCl, 10 mm MgCl_2_, 2 mm DTT, 1 mm ATP, 1 mm PMSF, 100 × Protease Inhibitor Cocktail (P2714; Sigma), and 100 × Phosphate Inhibitor Cocktails I and II (Sigma, P2850 and P5726, respectively)] at 30 °C for 30 min. The reactions were initiated by adding 50 μL of either the cotton fiber cell extract as described above or BSA protein as a negative control and terminated by adding 10 μL of 6 × SDS/PAGE sampling buffer. The samples were boiled, separated by 12% SDS/PAGE, and examined by western blot analysis. Mass spectrometric analysis of the phosphorylation sites was performed using a 4800 MALDI‐TOF/TOF analyzer (Applied Biosystems, Framingham, MA, USA) as previously described 23.

### *In vitro* degradation assay

An *in vitro* degradation assay was performed as described with minor modifications 24. Approximately 1 g of frozen cotton fiber was ground in liquid nitrogen and suspended in a cell‐free degradation assay buffer [25 mm Tris‐HCl (pH 7.5), 10 mm MgCl_2_, 5 mm DTT, 10 mm NaCl, and 10 mm ATP], followed by centrifugation to remove the cell debris. Equal amounts (300 μg) of total protein were added to the recombinant protein samples (5 μg of purified His‐tag wt and mutant GhPK6 proteins) in a total reaction volume of 110 μL. The reaction mixtures were incubated in the dark, and 20 μL of the reaction mixture was removed at different time points (0, 20, 40, 60, or 120 min) and placed in new tubes containing 4 μL of 6 × SDS/PAGE sample buffer to stop the degradation process. Then, the samples were boiled for 3 min, separated by 12% SDS/PAGE and subjected to western blot analysis using an anti‐His monoclonal antibody (70796; Novagen).

### Statistical analyses

All the experiments were repeated with at least three biological and technical replicates. Student's *t*‐test was used to determine the significance of the effect of site mutations on GhPK6 activity and yeast growth; these tests were performed with the spss 16.0 statistical software package (IBM, Chicago, IL, USA). Statistical significance was defined as the 0.05 (*) and 0.01 (**) levels of probability.

## Results

### The cotton cytosolic pyruvate kinase GhPK6 was phosphorylated at serine 215 and 402 sites

**Figure 1:**
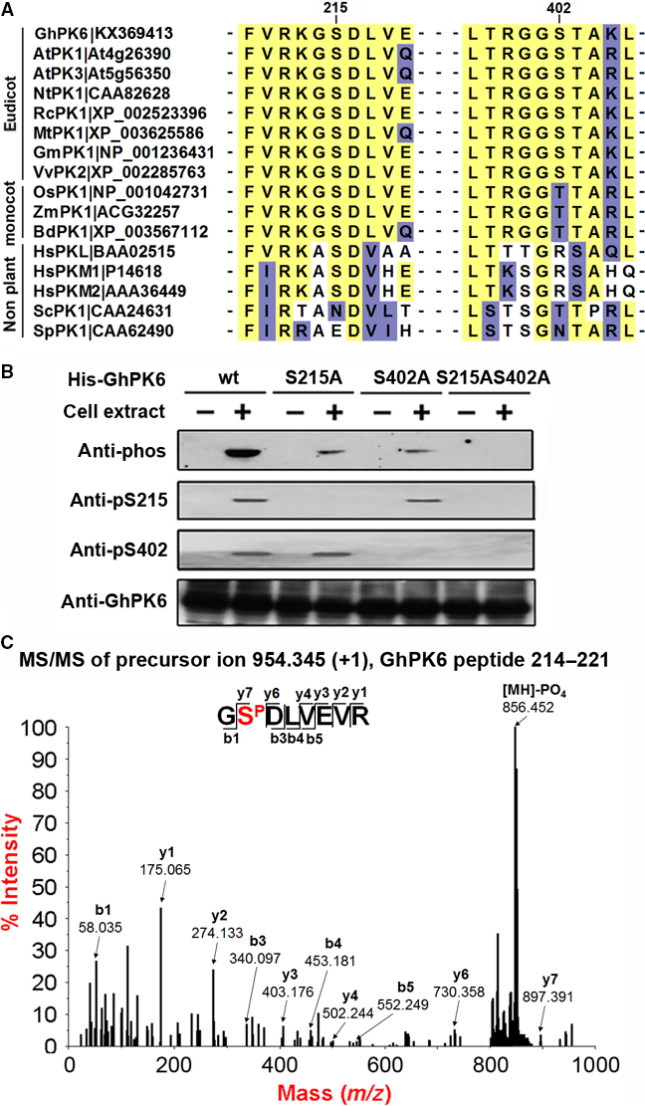
GhPK6 was phosphorylated at serines 215 and 402. (A) Alignment of the serine 215 and 402 phosphorylation domain of GhPK6 with PK proteins from other plants, animals, and yeast. The conserved amino acid sites are highlighted in yellow. (B) Analyses of the phosphorylation sites of GhPK6. The purified His‐tagged GhPK6 (wt) and its S215A, S402A and S215A, S402A mutant proteins were incubated either with or without cotton fiber cell extracts (+ and −, respectively). The serine 215 and serine 402 phosphorylation levels were then analyzed using western blotting. (C) MS/MS spectra of the phosphopeptide GS^P^DLVEVR. The phosphopeptide spectra were acquired manually through inputting the peptide mass in the precursor mass window of the 4800 MALDI‐TOF/TOF™ Analyzer.

The serine residues 220 and 407 were previously identified as phosphorylation sites in soybean PKc protein 16. Amino acid sequence alignment revealed that the two phosphorylation sites and their surrounding sequences (FVRKGS^215^DLVE and VLTRGGS^402^TAKL) are conserved in the cotton cytosolic PK GhPK6 (Fig. 1A). Western blotting using antibodies that detected pan‐phosphorylation as well as site‐specific phosphorylation both indicated that purified recombinant His‐GhPK6 protein could be phosphorylated at serines 215 and 402 by a cotton fiber cell extract (Fig. 1B). The phosphorylation of His‐GhPK6 protein at serine 215 was successfully identified using tandem mass spectrometric analysis (Fig. 1C). Furthermore, the observed phosphorylation was abated when the two serine sites were mutated to alanine residues (Fig. 1B). These results indicated that the cotton cytosolic PK GhPK6 was phosphorylated at serines 215 and 402.

### Enzyme activity of GhPK6 was regulated by phosphorylation

**Figure 2:**
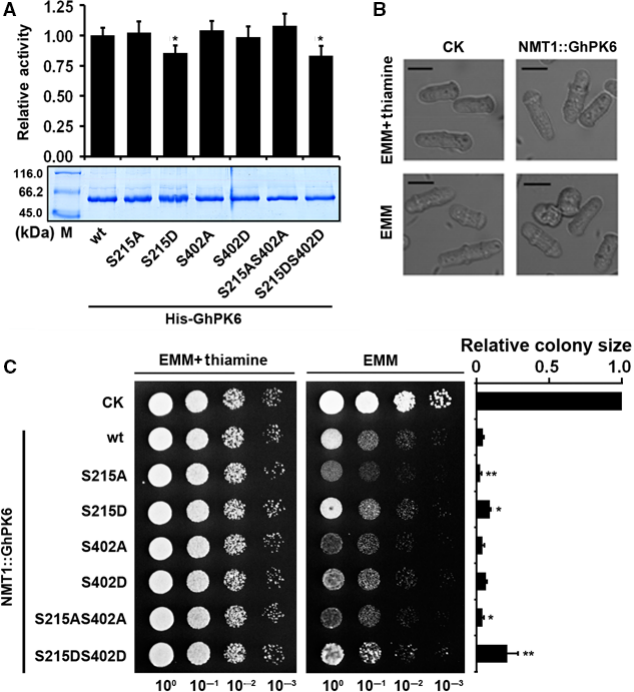
Phosphorylation inhibited the activity of GhPK6 (A) Relative enzyme activity of the purified His‐tagged GhPK6 (wt) and the S215A, S215D, S402A, S402D, S215A, S402A, and S215D, S402D mutant proteins. The ratios of *V*_max_ of the mutant proteins versus wt protein were calculated. SDS/PAGE of the purified proteins was shown at the bottom. Error bars indicate SE. (B) Microscopic image of fission yeast SPQ‐01 expressing (without thiamine) or not expressing (with thiamine) GhPK6 compared to the yeast transformed with empty vector pESP2M (CK). Bar = 10 μm. (C) Phenotypes of the fission yeast SPQ‐01‐expressing wt GhPK6 protein and the phosphorylation site mutants of GhPK6. Yeast cells expressing the pESP2M empty vector were used as a negative control. The measurement and statistical analysis of the average colony size of these yeast strains in 10^−2^ dilution are shown on the right. Error bars indicate SE. *P < 0.05; **P < 0.01.

To determine the physiological functions of phosphorylation, the effect of phosphorylation on GhPK6 enzyme activity was first investigated by incorporating site‐specific mutations. The mutation of serine 215 or serine 402 to alanine did not change the enzymatic activity; furthermore, mutation of serine 402 to the phosphomimetic aspartic acid also imparted no effect on enzymatic activity (Fig. 2A). However, enzyme activity was significantly reduced when serine 215 was mutated to aspartic acid (Fig. 2A). Under the control of the NMT1 promoter, the GhPK6 protein was expressed in fission yeast when thiamine was excluded from the growth medium 20, resulting in a strong inhibition of cell growth that was directly reflected in the growth curve, cell length, and colony size (Figs 2B and S2). Overexpression of the serine‐215‐to‐alanine mutant GhPK6 inhibited yeast growth more than that of the wild‐type GhPK6, whereas the mutation of serine 215 to aspartic acid significantly relieved this inhibition (Fig. 2C). Mutation of serine 402 to either alanine or aspartic acid alone did not significantly change the colony size of the fission yeast, however, mutating both serine 215 and serine 402 to aspartic acid relieved the inhibition of yeast growth more than the serine 215 single mutation did (Fig. 2C). These results collectively indicated that the activity of the cotton cytosolic PK GhPK6 was strongly regulated by phosphorylation at the serine 215 site and weakly regulated by serine 402 phosphorylation.

### Phosphorylation of GhPK6 regulated its stability

**Figure 3:**
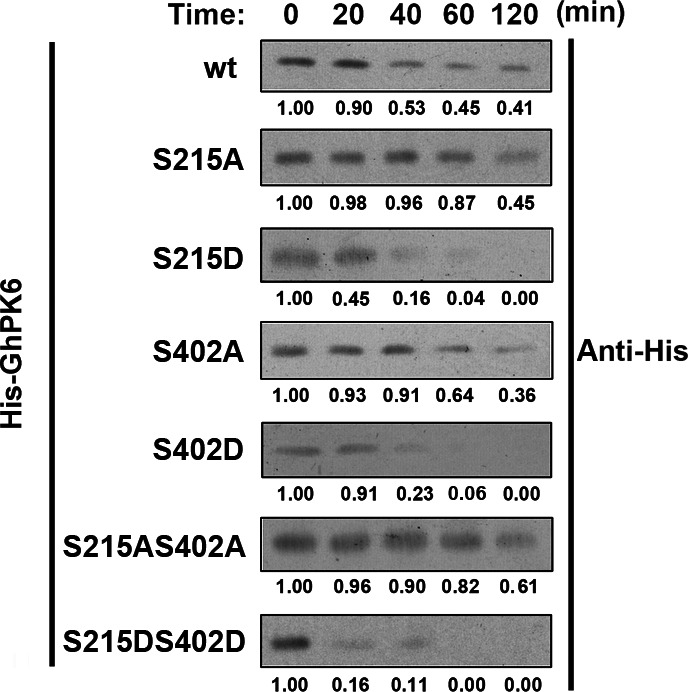
Phosphorylation promoted the degradation of GhPK6. Western blot analyses showing that the S215A, S402A and S215A, S402A phosphorylation site mutations in recombinant His‐GhPK6 proteins were more stable than the wild‐type (wt) and the S215D, S402D and S215D, S402D mutant His‐GhPK6 proteins. The bands were quantified by densitometry and were labeled as a percentage.

The effect of GhPK6 phosphorylation on its stability was also investigated. The half‐life of the GhPK6 protein is approximately 60 min. The mutation of serine 215 to alanine increased its half‐life to more than 120 min, whereas mutation of the same residue to aspartic acid decreased its half‐life to less than 20 min (Fig. 3). Similar half‐life changes were also observed when serine 402 of GhPK6 was mutated to either alanine or aspartic acid, although the differences were less obvious (more than 60 min and less than 40 min, respectively). When the two sites were both mutated to either alanine or aspartic acid, the stability change was more intense but similar to the effects observed with the single mutation at serine 215 (Fig. 3), suggesting that phosphorylation at serine 215 affects the protein stability of GhPK6 to a greater extent than phosphorylation of serine 402. These results were consistent with the results of the yeast overexpression phenotype, suggesting that the observed change in GhPK6 activity caused by phosphorylation/dephosphorylation mimetics might be achieved by either increasing or decreasing the protein degradation rate, respectively.

### Phosphorylation‐mediated degradation of GhPK6 regulated its protein abundance during the cotton fiber elongation process

**Figure 4:**
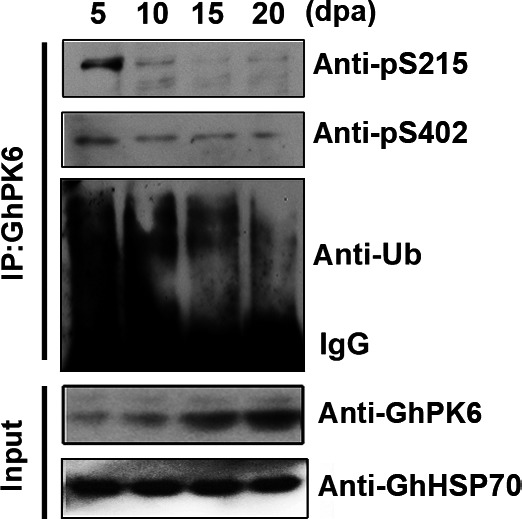
Phosphorylation promoted the ubiquitination of GhPK6. Immunoprecipitation of the cotton total protein using an anti‐GhPK6 antibody as bait followed by western blotting showing that both the ubiquitination and phosphorylation levels of GhPK6 protein gradually decreased at four time points during the cotton fiber elongation process. The cotton GhHSP70 protein served as a loading control.

The effect of GhPK6 phosphorylation on its protein stability was further analyzed in elongating cotton fiber cells. Immunoprecipitation using a GhPK6‐specific antibody and western blot indicated that phosphorylation of GhPK6 at serines 215 and 402 was most prominent at 5 days post anthesis (dpa), while the poly‐ubiquitination of GhPK6 was also most intense at 5 dpa (Fig. 4). The phosphorylation and ubiquitination of GhPK6 both gradually decreased after 5 dpa. In contrast, the protein abundance of GhPK6 gradually increased from 5 to 20 dpa (Fig. 4). These results, in combination with the finding that the protein‐to‐mRNA ratio of GhPK6 gradually increased during cotton fiber elongation process (Fig. S3) 15, strongly suggested that phosphorylation at serines 215 and 402 could promote the ubiquitin‐dependent degradation of GhPK6 to finely regulate its protein abundance in elongating cotton fiber cells.

## Discussion

Protein phosphorylation is one of the most relevant and ubiquitous post‐translational modifications. As a reversible modification, phosphorylation affects both the folding and function of proteins as well as regulates the activities of many important metabolic enzymes in plants. In tobacco, the nitrogen assimilation‐related nitrate reductase is phosphorylated at serine 521, which inhibits its activity by promoting an interaction with 14‐3‐3 proteins 25. In developing castor oil seeds, different subunits of phosphoenolpyruvate carboxylases (PEPC) are phosphorylated at multiple sites, among which the phosphorylation at serine 425 of bacterial‐type PEPC attenuates its catalytic activity 26. In maize, sucrose synthase is phosphorylated at serine 15 site on the N terminus, which could significantly increase the sucrose cleavage activity 27. In this study, we demonstrated that phosphorylation at serines 215 and 402 on the cotton PK GhPK6 regulated the protein abundance and enzymatic activity in elongating cotton fiber cells, providing another vivid example of phosphorylation‐based regulation of plant metabolic enzymes.

Previous studies have collectively revealed that yeast PK plays a central regulatory role in the metabolism of proliferating yeast cells by maintaining the balance between energy production, ROS clearance, and amino acid biosynthesis 28, 29. Sequence alignment indicated that GhPK6 shares 40.5% amino acid similarity to the fission yeast PK SpPYK1 (NCBI accession No: CAA62490) (Fig. S4). Overexpression of GhPK6 protein could increase the total PK activity of yeast cells, resulting in the disturbance of metabolic homeostasis and inevitably inhibit the normal growth of yeast cells (Figs 2B and S2). Because the effect of PK on the metabolism and growth of yeast cells is dosage‐dependent 28, overexpression of the more stable GhPK6 protein (i.e., serine‐215‐to‐alanine mutant) will have a stronger inhibitory effect on yeast growth (Fig. 2C).

**Figure 5:**
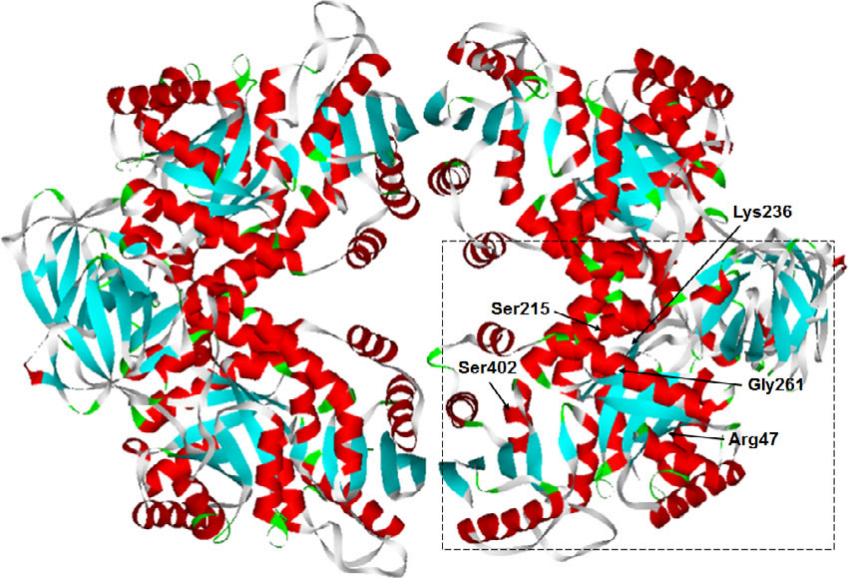
Three‐dimensional structure of the GhPK6 protein The GhPK6 monomer forms an active tetramer *in vivo*. The two phosphorylation serine sites (Ser215 and Ser402) and amino acid residues important for transition state stabilization (Lys236) and substrate binding (Arg47 and Gly261) were all marked with arrows in one of the monomers (dash box).

Our results indicated that only the phosphorylation‐mimetic mutation at the serine 215 site could significantly reduce the enzymatic activity of purified recombinant GhPK6 protein (Fig. 2A). However, the colony size of fission yeasts expressing GhPK6 proteins harboring phosphorylation/dephosphorylation‐mimetic mutations at the serine 215 site were both significantly changed (Fig. 2C). The discrepancy of these two results is understandable because the enzymatic activity of purified GhPK6 protein is only affected by its structural characteristics. Structure simulations of the GhPK6 protein revealed that the serine 215 site is adjacent to lysine 236 and glycine 261 (Fig. 5). The lysine 236 residue is required for transition state stabilization of the enzyme tetramer, and glycine 261 is involved in substrate binding 30, 31. Mutation of the serine 215 site to a more acidic residue (i.e., aspartic acid) would change the electrochemical environment surrounding the two activity‐related residues, whereas mutation of serine 215 to alanine as well as mutations of the distantly located serine 402 site would not have this impact.

Our results also revealed that phosphorylation of cotton PK GhPK6 promoted its ubiquitination‐dependent degradation during the early stages of the cotton fiber elongation process (Fig. 4). Previous results indicated that inhibition of GhPK6 expression increased the length of mature cotton fibers 15, thus the phosphorylation‐mediated degradation of GhPK6 may have a similar effect in promoting the elongation of cotton fibers. In conjunction with the finding that acetylation‐mediated degradation of PKM2 promotes cancer cell growth in rapidly proliferating animal tumors 9, our results strongly suggested that post‐translational modifications of PK are important for regulating fast cell growth in both animals and plants. In the future, we could further characterize the corresponding kinases and phosphatases that regulate the phosphorylation status of the two serine sites and develop a molecular model depicting the dynamic regulatory mechanism of GhPK6 activity, its protein abundance, and its effects on the cotton fiber elongation rate.

## Author contributions

BZ and JL conceived and designed research, and wrote the manuscript. BZ conducted experiments and analyzed data. Both authors read and approved the manuscript.

## Supporting information


**Fig. S1.** Purification of the two phosphorylation site‐specific antibodies.

**Fig. S2.** Phenotypes of the fission yeast SPQ‐01 overexpressing GhPK6.

**Fig. S3.** mRNA and protein expression of GhPK6 in elongating cotton fibers.

**Fig. S4.** Amino sequence alignment of GhPK6 with fission yeast pyruvate kinase SpPYK1.

**Table S1.** Primers used in this study.

**Table S2.** Peptide sequences for antibody preparation.
