# Supplementary figures and images for: Serine phosphorylation of the cotton cytosolic pyruvate kinase GhPK6 decreases its stability and activity
Source: FEBS Open Bio. 2017 Jan 25;7(3):358–66. doi: 10.1002/2211-5463.12179 (PMC5337898; doi:10.1002/2211-5463.12179)

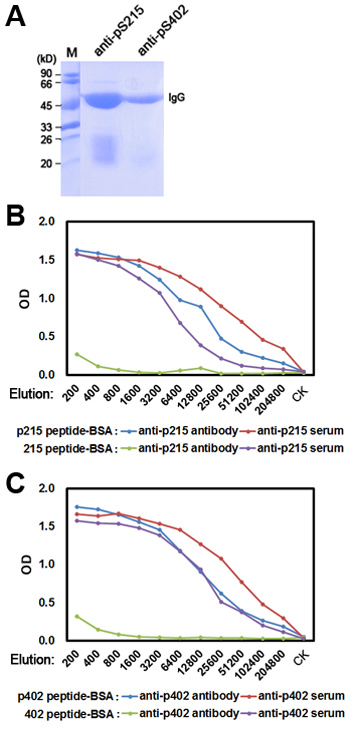

Supplement: Supplementary file 1 — Fig. S1. Purification of the two phosphorylation site‐specific antibodies. [file FEB4-7-358-s001.tif]

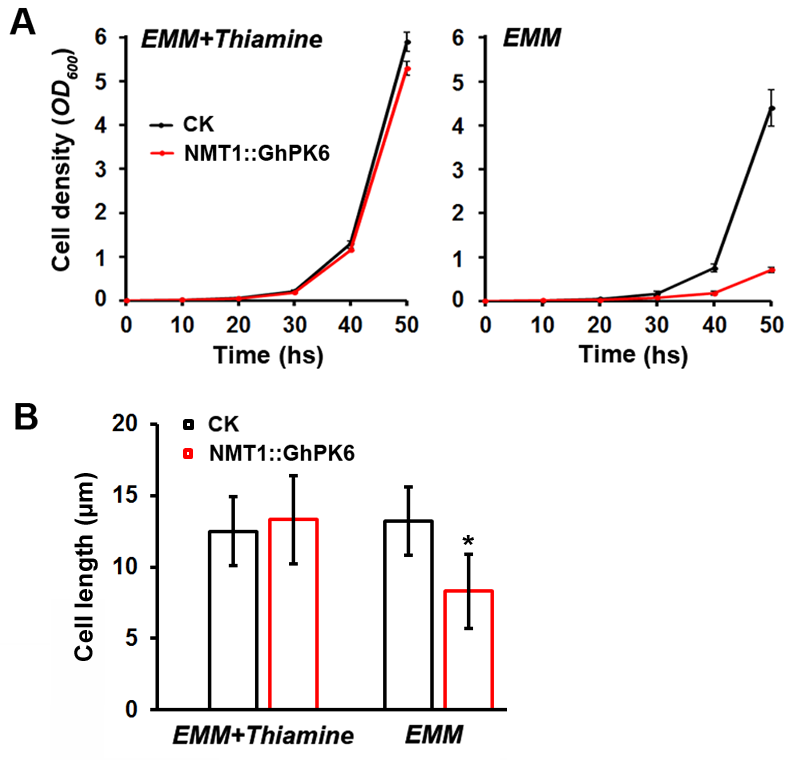

Supplement: Supplementary file 2 — Fig. S2. Phenotypes of the fission yeast SPQ‐01 overexpressing GhPK6. [file FEB4-7-358-s002.tif]

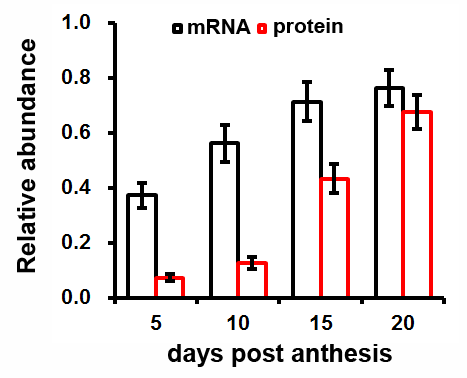

Supplement: Supplementary file 3 — Fig. S3. mRNA and protein expression of GhPK6 in elongating cotton fibers. [file FEB4-7-358-s003.tif]

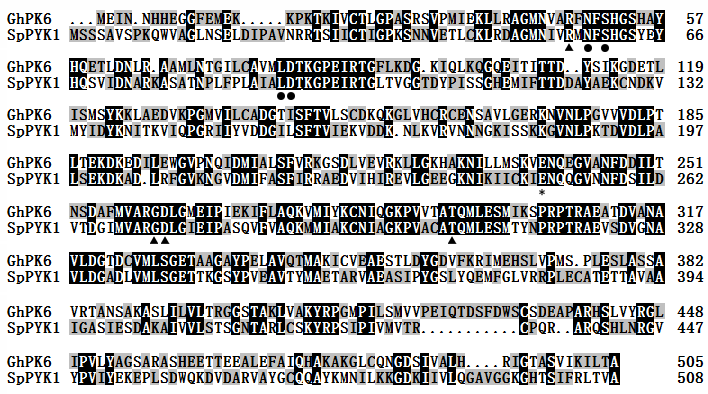

Supplement: Supplementary file 4 — Fig. S4. Amino sequence alignment of GhPK6 with fission yeast pyruvate kinase SpPYK1. [file FEB4-7-358-s004.tif]
